# Guaiphenesin-ketamine-xylazine infusion to maintain anesthesia in mules undergoing field castration

**DOI:** 10.1186/s13028-017-0335-7

**Published:** 2017-10-11

**Authors:** Cecilia Vullo, Augusto Carluccio, Domenico Robbe, Marina Meligrana, Linda Petrucci, Giuseppe Catone

**Affiliations:** 1 School of Pharmacy, University of Camerino, Via Madonna delle Carceri, 62032 Camerino, Italy; 2 School of Veterinary Medicine, University of Teramo, Località Piano D’Accio, 64100 Teramo, Italy; 3 School of Biosciences and Veterinary Medicine, University of Camerino, Via Circonvallazione, 62022 Matelica, Italy; 4 School of Veterinary Medicine, University of Perugia, Via San Costanzo, 06126 Perugia, Italy

**Keywords:** Field castration, Guaiphenesin, Ketamine, Mules, Thiopental, Xylazine

## Abstract

**Background:**

In order to determine whether a combination of guaiphenesin, ketamine and xylazine can induce safe and satisfactory anaesthesia in mules undergoing field castration, eight healthy adult intact male mules were employed. They were premedicated with intravenous (IV) xylazine (1.3 mg/kg); an additional dose of xylazine (0.3 mg/kg IV) was administered in case of inadequate depth of sedation. Anaesthesia was induced with IV thiopental (6 mg/kg). The quality of sedation and induction was recorded. Anaesthesia was maintained with an infusion of guaiphenesin (50 mg/mL), ketamine (2 mg/mL) and xylazine (1 mg/mL) (GKX). The spermatic cord of each testis was infiltrated with 5 mL of 2% lidocaine. During anaesthesia heart rate (HR), respiratory rate (RR), rectal temperature (RT) and haemoglobin oxygen saturation (SpO_2_) were measured every 5 min. The data were analysed with simple one-way analysis of variance (ANOVA). A *P* value < 0.05 was considered statistically significant. Time of anesthesia, time of surgery and time of recovery were recorded.

**Results:**

Only one mule required an additional dose of xylazine to achieve a satisfactory depth of sedation. Thiopental at the dose of 6 mg/kg IV resulted in smooth induction and lateral recumbency in all animals. GKX provided adequate anaesthesia to perform castration in all mules. Muscle relaxation was deemed adequate and physiological variables remained stable and within references values during the anaesthesia and did not change in response to surgical stimulation. Time (mean ± standard deviation) from the end of the infusion to sternal recumbency and time from sternal recumbency to standing were 27.7 ± 4.6 and 30.1 ± 7.7 min, respectively.

**Conclusions:**

The combination of xylazine, thiopental and GKX provides satisfactory short-term anaesthesia in mules undergoing field castration.

## Background

The mule (*Equus mulus*) is a hybrid between a male donkey and a female horse. Physiologically, mules look more like horses, although they are not identical. They may range in appearance and temperament depending on the type of horse used in breeding [1]. Despite a number of studies available in the literature regarding anaesthesia in horses, donkeys and ponies, few studies have been conducted on mule anaesthesia [2–4]. The results of these studies have been applied to mules, resulting in inadequate anaesthesia because there are several anatomical, physiological and pharmacological differences between horses and mules [5]. Total intravenous anaesthesia (TIVA) is usually the option of choice under field conditions, and it has become a popular technique in horses because of its advantages over inhalational anaesthesia, including decreased cardiorespiratory depression [6]. Many anaesthetic techniques are suitable for field conditions, including the combination commonly referred to as “triple drip”, a mixture of a dissociative anaesthetic (ketamine), an adrenergic alpha 2 receptor agonist (xylazine, romifidine or detomidine), and a centrally acting muscle relaxant (guaiphenesin) [7]. This solution is infused at a predetermined rate or titrated to effect [8, 9]. This approach yields excellent muscle relaxation and mild to moderate analgesia. Although hypoxaemia can occur, serious cardiorespiratory depression is rarely reported in short procedures [10, 11]. However, the use of this technique in horses is limited to procedures lasting no more than 60–90 min [9]. Similar to horses, castration is performed under general anaesthesia in mules, for the safety of both the veterinarian and the mule and in the interest of animal welfare. The purpose of this study was to determine whether the combination of guaiphenesin, ketamine and xylazine induces safe and satisfactory total intravenous anaesthesia in mules undergoing field castration.

## Methods

The study was approved by the Institutional Animal Ethics Committee, University of Teramo. Eight healthy adult intact male mules, American Society of Anesthesiologists (ASA) physical status I or II, aged 4–6 years and weighing 380–450 kg were employed. Exclusion criteria included ASA physical status ≥ 3, intractable behavior, neurologic or neuromuscular disease, skin infection at the site of the surgery. Body weight was estimated based on the body length and chest girth of the mules [12]. The animals were consecutively anaesthetised by the same team of two anaesthetists to perform field castration with the aim of reducing the typical exuberance of male animals and improving work performance, specifically the transport of firewood, for which these animals are widely used in the Italian regions of Marche and Abruzzo. Baseline rectal temperature (RT), heart rate (HR), and respiratory rate (RR) were recorded. RT was measured using a digital thermometer, HR by auscultation using a stethoscope, and RR by observing thoracic excursion. Before anaesthesia, a 14-gauge, 13-cm catheter was placed in the external jugular vein, across a bleb of lidocaine previously injected subcutaneously. Mules were premedicated with 1.3 mg/kg of xylazine (Rompun^®^; Bayer, Italy) intravenously (IV) and sedation was considered adequate based on lowering of the head, drooping of the lower lip and drooping of the ears, graded on a 4-point sedation scale (score 0, poor: fully responsive to environment, lips apposed, no lowering of head, no drooping of the ears; score 1, mild: still responsive to environment, slight separation of the lower lip, slight lowering of the head, slight drooping of the ears; score 2, good: no response to environment, separation of the lower lip, lowering of the head, drooping of the ears; score 3, heavy: no response to environment, extreme lip separation, pronounced loss of postural tone and ataxia, pronounced separation of the ear tips). Additional xylazine (0.3 mg/kg IV) was administered when the mules were inadequately sedated. Anaesthesia was then induced with 6 mg/kg IV of thiopental (Pentothal sodium^®^; Intervet, Italy) within 5 min from premedication. A 4-point induction score was used to evaluate the quality of induction (score 0, poor: ataxia, excitement without falling down; score 1, sufficient: ataxia, fall with paddling and attempts to stand up; score 2, good: no ataxia, able to move 1 or 2 steps with no paddling after falling down; score 3, very good: no ataxia, smoothly falling down to the ground). An additional dose of thiopental (1 mg/kg IV) had been scheduled were the induction to prove insufficient. Once the mules were recumbent, the infusion of guaiphenesin (Knockout^®^; ACME, Italy), ketamine (Ketavet 100^®^; Intervet, Italy) and xylazine (GKX) was started to maintain general anaesthesia, beginning with one drop/s, and decreasing or increasing the infusion rate based on monitoring of eye signs, muscle relaxation of the neck, RR and pattern, and response to surgical stimulation, but not exceeding three drops/s, using a rate flow regulator set (B. BRAUN’S^®^ rate flow 20 drops/mL) with a range of 1.5 to 9 mL/min. The solution was created by adding 1 g of ketamine and 500 mg of xylazine to a 500-mL bag of 5% guaiphenesin to obtain a solution of 50 mg/mL guaiphenesin, 2 mg/mL ketamine and 1 mg/mL xylazine.

After the induction of anaesthesia, the head and neck were extended to maintain a patent airway. The spermatic cord of each testis was infiltrated with 5 mL of 2% lidocaine (Lidocaina 2%; Esteve^®^, Italy) to achieve intratesticular analgesia. The time of infiltration was considered the start time of the surgery. In each animal the castration was performed by the same team of two surgeons using Serra’s emasculator and an open technique was used for all mules in order to promote postoperative drainage. During anaesthesia HR, RR, RT and haemoglobin oxygen saturation (SpO_2_, Nellcor™ Portable SpO_2_, Covidien), with the clip of the pulse oximetry probe applied to the tongue, were measured every 5 min (Table 1).

**Table 1:** Baseline and following xylazine-thiopental and GKX infusion parameters in mules (*n* = 8)

Time-point	− 60 min	0 min	5 min	10 min	15 min	20 min
HR	44.63 ± 1.2	44.75 ± 2.6	44 ± 3.02	43.7 ± 1.98	44.5 ± 2.07	43.7 ± 2.7
RR	24.8 ± 0.6	22.5 ± 2.07	22.5 ± 2.32	22.5 ± 2.5	22.75 ± 1.03	23 ± 1.85
RT	38 ± 0.5	37.3 ± 0.49	36.95 ± 0.42	36.78 ± 0.31	36.66 ± 0.25	36.61 ± 0.24
SpO_2_	NR	NR	97.62 ± 1.06	97.75 ± 0.70	97.75 ± 0.88	97.37 ± 1.06

Values are given as the mean ± standard deviation

*NR* not reported

The blood pressure and blood gas analyses weren’t monitored due to the unavailability of the equipment. The anesthesia time (time from induction to the end of the infusion), the time required to perform the aseptic preparation, the time to carry out the surgery (spermatic cord infiltration and surgery), the time from the end of the infusion to sternal recumbency and the time from sternal recumbency to standing (recovery time) were recorded (Table 2).

**Table 2:** Anesthesia, surgery and recovery time in mules (*n* = 8)

Parameter	Mean values ± SD
Anesthesia time (min)	32.8 ± 4.3
Aseptic preparation (min)	5.7 ± 2.9
Surgery time (min)	20.8 ± 2.8
Time to sternal recumbency (min)	27.7 ± 4.6
Time to standing (min)	30.1 ± 7.7
Recovery time (min)	57.8 ± 12.3
Number of attempts to stand	1

Values are given as the mean ± standard deviation

### Statistical analysis

The data recorded and reported in Table 1 were analysed using a simple one-way analysis of variance (ANOVA) and a *P* value < 0.05 was considered significant. The data are reported as the mean ± standard deviation (SD).

## Results

The eight mules had a mean age ± SD of 4.8 ± 0.75 years. Their average estimated weight was 415 ± 49.5 kg. The sedation scores achieved were 0 in one animal, 1 in one animal, 2 in five animals and 3 in one animal. An additional dose of 0.3 mg/kg of IV xylazine was administered to the mule that had a poor sedation score. Induction scores were 2 in six animals and 3 in two animals. As no animal presented an induction score of 0 or 1, it was not necessary to administer an extra dose of thiopental. In all animals, the fall was characterized by a lack of hypertonicity of the pelvic limbs. The infusion of the GKX combination was initiated at one drop/s immediately once the mules fell down. When the surgery started, eye signs, muscle relaxation of the neck, RR and pattern, and response to surgical stimulation were monitored to calibrate the infusion rate. Only one animal showed signs of nystagmus at the time of infiltration of the spermatic cord and two animals showed a slight degree of contraction of the hind limb opposite to the recumbency, prompting an increase in the infusion rate to two drops/s until these signs disappeared. There were no significant differences in HR (44 ± 2.3), RR (22.6 ± 1.9), or RT (36.7 ± 0.3) when compared to the baseline values. The mean SpO_2_ was 97.6% ± 0.9 (Table 1). The surgery was completed in all the animals, and there were no anaesthetic or surgical complications. The time required to perform aseptic preparation was 5.7 ± 2.9 min. The surgery time (spermatic cord infiltration and surgery) was 20.8 ± 2.8 min. The time from induction to the end of the infusion (anesthesia time) was 32.8 ± 4.3. Times from the end of the infusion to sternal recumbency were 27.7 ± 4.6 min and times from sternal recumbency to standing were 30.1 ± 7.7 min. Therefore, the recovery time (time from the end of the infusion to the time of standing) was 57.8 ± 12.3 min, which is not considered prolonged, as mules remain recumbent until they are able to achieve a standing position at the first attempt. The animals required only one attempt to come to a standing position (Table 2).

## Discussion

Given that previous similar studies on mules and donkeys used a sample of six animals [13, 17], a total of 8 was considered sufficient to carry out the present study and to find relevant results. As in former studies, the method used to evaluate the weight of the mules was extrapolated from a method used in horses and therefore may not be entirely adequate for use in this species. The 1.3 mg/kg dose of IV xylazine was adequate in 5 out of six animals. This is consistent with a previous study [13], demonstrating that mules require approximately 50% more xylazine (and probably other alpha 2 agonists) than either donkeys or horses [14]. The inadequate sedation that was observed in one mule is probably related to the fact that the mule presented with a more nervous temperament. We therefore performed the IV premedication immediately before the catheter was positioned. The time required for the catheter placement determined the loss of the xylazine’s effect, as the half-life of this drug is 15 min shorter (32 min) in mules than in horses (47 min) [5].

The 6 mg/kg dose of thiopental was adequate, and no animal showed ataxia with excitement without falling down or fell down while paddling and trying to stand up. A short apnea or cardiorespiratory depression after thiopental administration is not unusual [15, 16]. However, in agreement with the results of a previous study in donkeys premedicated with 1 mg/kg of xylazine and induced with 10 mg/kg of thiopental [17], in our study we never observed significantly different values of HR and RR from baseline.

We chose thiopental for the induction to avoid incomplete muscle relaxation during the surgery and the ataxia that is frequently observed during recovery with ketamine induction [7] and for its rapid onset of the action (30–60 s) and brief duration of anesthesia (10–20 min) [18].

The GKX combination allowed the animals to be maintained at stage III, plane 2 of general anaesthesia. The increase in the GKX infusion rate, from one drop/s to two drops/s, guaranteed the rapid achievement of the surgical plane in three animals that showed signs of lightening anaesthesia. Various infusion rates for GKX have been previously reported for induction and maintenance in donkeys. In a study by Taylor et al. three different combinations of GKX were used to induce and maintain anaesthesia for 45 min in donkeys and the authors concluded that only the combination of 2.0 mg/mL ketamine, 0.5 mg/mL xylazine, and 50 mg/mL guaiphenesin produced satisfactory anaesthesia without significant respiratory depression and would induce safe and effective anaesthesia under field conditions [19]. We have come to the same conclusions, although we used a higher concentration of xylazine for maintenance of the anaesthesia, since mules are generally less responsive to xylazine than donkeys.

Coelho et al. evaluated the cardiorespiratory and biochemical effects of guaiphenesin (50 mg/mL), ketamine (2.0 mg/mL) and xylazine (0.5 mg/mL) anaesthesia for 1 h in donkeys induced with diazepam (0.05 mg/kg) and ketamine (2.2 mg/kg). The mixture was administered as a constant rate infusion (2.0 mL/kg/hr) using an infusion pump. They concluded that this protocol induced significant hypoxaemia but no other cardiorespiratory or metabolic changes [20]. In our study, we never observed a decrease in SpO_2_, but this was likely related to the short surgical time and, consequently, the short period of infusion. Furthermore, the GKX mixture allowed anaesthesia characterized by excellent muscle relaxation, good analgesia and an uneventful, smooth and quiet recovery, with a mean recovery time of 57.8 ± 12.3 and only one attempt to successfully achieve a standing position. Nevertheless, it is important to consider that the muscle relaxation could also be attributed to the effects of thiopental. However, since thiopental is classified as a short-acting barbiturate, it is likely that we took advantage of its beneficial effects solely during the aseptic preparation and the initial stages of the surgery.

A significant decrease in RT has been observed during general anaesthesia in horses [21] but did not occur in the present study. This may also be attributed to the short duration of anaesthesia and to the good local weather.

We chose not to administer opioids or non-steroidal anti-inflammatory drugs in our study because the analgesia was provided by the infusion of ketamine and xylazine and by the infiltration of lidocaine in the spermatic cord. Furthermore, the lack of preemptive analgesia allowed us to assess whether the administration of GKX alone could be able to guarantee adequate analgesia.

## Conclusions

These results suggest that the administration of a GKX combination resulted in anaesthesia characterized by satisfactory muscle relaxation and excellent recovery, without significant differences in HR, RR or RT compared to baseline values. In conclusion, the GKX combination can be used safely for short-term TIVA in mules undergoing castration in field conditions premedicated with xylazine and induced with thiopental. However, further investigation of cardiovascular parameters and blood gas analyses should be undertaken to obtain more accurate information about the effects of this anaesthetic combination.

## Abbreviations

ANOVA: analysis of variance
ASA: American Society of Anesthesiologists
GKX: guaiphenesin-ketamine-xylazine
HR: heart rate
IV: intravenous
RR: respiratory rate
RT: rectal temperature
SD: standard deviation
SpO_2_: haemoglobin oxygen saturation
TIVA: total intravenous anaesthesia

## References

[1] Matthews NS, Taylor TS. Anesthetic management of donkeys and mules. In: Steffey EP (ed.). Recent advances in anesthetic management of large domestic animals. Ithaca: International Veterinary Information Service; 2000. Document No. A0607.0700. http://www.ivis.org/advances/Steffey_Anesthesia/matthews_donkeys/chapter.asp?LA=1. Accessed 15 Feb 2017.

[2] Matthews NS, Taylor TS, Hartsfield SM, Williams JD (1992). A comparison of injectable anaesthetic regimens in Mammoth asses. Eq Vet J.

[3] Matthews NS, Taylor TS, Skrobarcek CL, Williams DJ (1992). A comparison of injectable anaesthetic regimens in mules. Eq Vet J.

[4] Matthews NS, Taylor TS, Hartsfield SM, Hayton WL, Jones DH (1994). Pharmacokinetics of ketamine in mules and mammoth asses premedicated with xylazine. Eq Vet J.

[5] Latzel ST (2012). Subspecies studies: pharmacokinetics and pharmacodynamics of a single intravenous dose of xylazine in adult mules and adult haflinger horses. J Eq Vet Sci.

[6] McMurphy RM, Young LE, Marlin DJ, Walsh K (2002). Comparison of the cardiopulmonary effects of anesthesia maintained by continuous infusion of romifidine, guaiphenesin, and ketamine with anesthesia maintained by inhalation of halothane in horses. Am J Vet Res.

[7] Staffieri F, Driessen B (2007). Field anaesthesia in the equine. Clin Tech Eq Pract..

[8] Taylor PM, Luna SPL, Sear JW, Wheeler MJ (1995). Total intravenous anaesthesia in ponies using detomidine, ketamine and guaiphenesin: pharmacokinetics, cardiopulmonary and endocrine effects. Res Vet Sci.

[9] El-Ghoul W, Zabady M, Saleh I (2004). Total intravenous anaesthesia in donkeys (*Equus asinus*): comparison of anaesthetic and cardiorespiratory effects of four anaesthetic drug combinations. Vet Med J.

[10] Young LE, Bartram DH, Diamond MJ, Gregg AS, Jones RS (1993). Clinical evaluation of an infusion of xylazine, guaiphenesin and ketamine for maintenance of anaesthesia in horses. Eq Vet J.

[11] Matthews NS, Peck KE, Maeley KL, Taylorr TS, Ray AC (1997). Pharmacokinetics and cardiopulmonary effects of guaiphenesin in donkeys. J Vet Pharmacol Ther.

[12] Carroll CL, Huntington PJ (1988). Body condition scoring and weight estimation of horses. Eq Vet J.

[13] Dar KH, Gupta AK (2016). Total intravenous anaesthesia in adult mules. Vet Anaesth Analg.

[14] Matthews NS, Taylor TS, Hartsfield SM (2005). Anaesthesia of donkeys and mules. Eq Vet Educ.

[15] Emami MR, Seifi H, Tavakoli Z (2006). Effects of totally intravenous thiopental anesthesia on cardiopulmonary and thermoregulatory system in donkeys. J Appl Anim Res.

[16] Matthews NS, Van Dijk P. Anesthesia and analgesia for donkey. In: Matthews NS, Taylor TS, editors. Veterinary care of donkeys. Ithaca: International Veterinary Information Service; 2004. Document No. A2902.0904. http://www.ivis.org/advances/Matthews/matthews/chapter.asp?LA=1. Accessed 18 Jul 2017.

[17] Abd-Almaseeh ZT (2008). Comparative anesthetic protocols: propofol and thiopental in xylazine premedicated donkeys. J Anim Vet Adv.

[18] McKelvey D, Hollingshead KW, McKelvey D, Hollingshead KW (2003). Anesthetic agents and techniques. Veterinary anesthesia and analgesia.

[19] Taylor EV, Baetge CL, Matthews NS, Taylor TS, Barling KS (2008). Guaiphenesin-ketamine-xylazine infusions to provide anesthesia in donkeys. J Eq Vet Sci.

[20] Coelho CMM, Moreno JCD, Goulart DS, Caetano LB, Soares LK, Coutinho GH, Alves GE, da Silva LA (2014). Evaluation of cardiorespiratory and biochemical effects of ketamine-propofol and guaifenesin-ketamine-xylazine anesthesia in donkeys (*Equus asinus*). Vet Anaesth and Analg.

[21] Malik V, Singh B (2007). Clinical and haematobiochemical studies on ketamine and its combinations with diazepam, midazolam and xylazine for general anaesthesia in horses. Indian J Vet Surg.

